# Through-slice dephasing for eddy current artifact reduction in bSSFP

**DOI:** 10.1186/1532-429X-14-S1-P271

**Published:** 2012-02-01

**Authors:** Ozan Sayin, John A Derbyshire, Elliot McVeigh, Daniel Herzka

**Affiliations:** 1 Biomedical Engineering, Johns Hopkins School of Medicine, Baltimore, MD, USA; 2 Translational Medicine Branch, DIR, National Heart, Lung and Blood Institute, NIH, Bethesda, MD, USA

## Summary

Eddy current effects can severely degrade image quality when using balanced steady-state free precession imaging with rapidly varying phase encode ordering schemes, which have common use in cardiac cine MRI. In this work, we explore and characterize a previously-proposed technique, through-slice dephasing, as the sole technique for eddy current artifact removal. We demonstrate that artifacts vary for different slice orientations yet they can be removed using the herein investigated technique.

## Background

**Figure 1:**
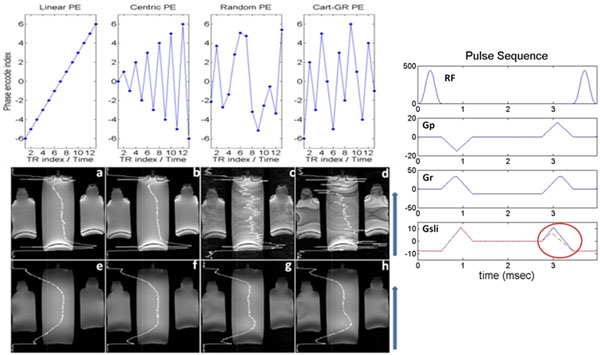
(Left panel, Top) Different phase encode strategies are displayed. (Left panel, Middle) a,b,c and d) Coronal scans with corresponding PE schemes. (Left Panel, Bottom) e,f,g and h) Same as a,b,c and d respectively, but with a through-slice dephasing of 60°. Blue arrow indicates PE direction. (Right Panel) bSSFP has fully balanced (zero net area) gradients (blue) Through-slice dephasing decreases the area of the slice selection rephasing gradient (red oval), leading to a small amount of intravoxel dephasing that eliminates the effects of rapidly fluctuating gradient fields.

Gradient pulses induce eddy currents in conductive components of scanners creating time-varying magnetic fields. For bSSFP, eddy currents create significant field fluctuations, strong enough to disturb the steady-state and introduce severe artifacts [1]. For linear phase encoding schemes, k-space lines are acquired consecutively, yielding a smooth variation of the induced fields over time. However, phase encode ordering schemes such as random, centric or golden-ratio [2,3] (Fig 1), employ large, irregular steps between successive k-space lines, causing varying field modulations and image artifacts. We explore through-slice dephasing [1,4] as a solution with minimal SNR penalties.

## Methods

Gd-doped water bottles were imaged on a 1.5T system (Avanto, Siemens Medical Systems, Erlangen, Germany) using the standard cardiac phased-array and spine coils. Max gradient amplitudes and slew rates were 33 mT/m and 130 mT/m/ms respectively. 2D bSSFP imaging was implemented using a hardware optimized gradient sequence design [5] (TR=3.0-3.5 msec). In addition to linear, centric and random PE orderings, a Cartesianized golden-ratio (Cart-GR) step ordering was tested. With Cart-GR, the resolution in the phase encode direction is a Fibonacci number (e.g. 377) and the step over the ky-grid between consecutively acquired PE lines is the previous one (e.g. 233). Cart-GR is guaranteed to sample exactly all the Cartesian grid without repetitions or missing lines for an arbitrary scan window [2]. All PE orderings were acquired using identical parameters (e.g. TE: TR/2, matrix: 377x384, FOV: 450 mm, flip angle: 35°). Through-slice dephasing was achieved by modifying the 0^th^ moment of the slice-selection dephasing gradient (Fig 1), creating symmetrical phase accrual at the end of each TR. Dephasing angles ranged between 0° and ±180° per TR. SNR and root-mean-square error (RMSE) values were computed using planimetry.

## Results

**Figure 2:**
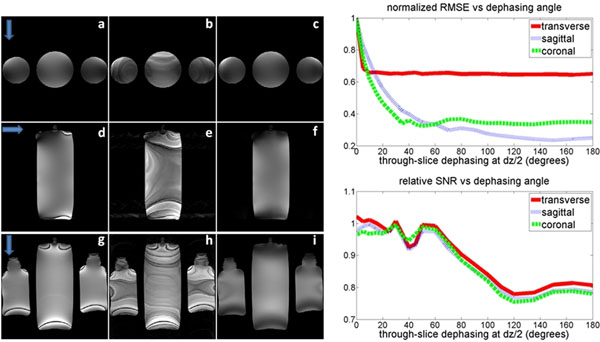
a) linear PE, b) Cart-GR PE , c) Cart-GR PE with through-slice (45° dephasing) transverse slice orientation. d), e) and f) are same as a), b) and c) respectively, with a sagittal slice. g), h) and i) are same as a), b) and c) respectively, with a coronal slice. Slice dephasing substantially improves image quality for Cart-GR PE. Blue arrows indicate PE direction. On the right, normalized RMSE (top) and SNR relative to the linear PE reference scan (bottom) values are plotted for Cart-GR scans. RMSE values are normalized to the fully balanced-SSFP Cart-GR scan for that particular orientation.

Figure 2 shows banding-like artifacts in all the Cart-GR images (especially the sagittal and coronal images). Artifacts are removed with ±45° through slice-dephasing. RMSE artifact reduction could be greatly improved with dephasing angles less than ±60° with SNR losses less than 10%.

## Conclusions

Through-slice dephasing is highly effective in suppressing eddy current induced artifacts in bSSFP imaging. Considering these artifacts appear substantial in Cart-GR and random PE scans, they are most likely caused by the zero order (spatially independent) EC field yielding an off-resonance shift over time. We propose that dephasing angles smaller than ±60°/TR provide sufficient suppression of EC artifacts with little SNR loss . More work is needed to determine effects of TSD on moving spins.

## Funding

This work was funded in part by Siemens Medical Solutions USA, Inc. and the American Heart Association, 11SDG5280025.
